# Glomus tumor of the duodenum: a rare case report

**DOI:** 10.1186/s40792-020-01084-5

**Published:** 2020-12-03

**Authors:** Shin Sasaki, Yuko Takami, Yoshiyuki Wada, Tomoki Ryu, Hajime Imamura, Hiroki Ureshino, Minako Fujiwara, Hideki Saitsu

**Affiliations:** 1 Department of Hepato-Biliary-Pancreatic Surgery, Clinical Research Institute, National Hospital Organization Kyushu Medical Center, 1-8-1 Jigyohama, Chuo-ku, Fukuoka, 810-8563 Japan; 2 Department of Pathology, National Hospital Organization Kyushu Medical Center, 1-8-1 Jigyohama, Chuo-ku, Fukuoka, 810-8563 Japan

**Keywords:** Duodenum, Glomus tumor, Submucosal tumor

## Abstract

**Background:**

Glomus tumors (GTs) are mesenchymal neoplastic lesions arising from the glomus bodies and generally occur in the fingers and toes. Gastrointestinal GTs are rare, and most of them originate from the stomach; however, GT arising from the duodenum is exceedingly rare.

**Case presentation:**

A 68-year-old man was admitted due to abdominal pain. Endoscopy showed a round, smooth, elevated mass in the second portion of the duodenum with central ulceration. Abdominal contrast computed tomography showed a hypervascular tumor measuring 26 mm in diameter in the second portion of the duodenum, and pancreatic invasion was suspected. Endoscopic ultrasonography of the lesion confirmed a hypoechoic mass arising from the fourth layer of the duodenal wall. A biopsy was performed for central ulceration, and immunochemical studies showed positive results for smooth muscle actin (SMA) and negative results for S100, C-Kit, and CD34. Leiomyoma or gastrointestinal stromal tumor was suspected and pancreatoduodenectomy was performed. The specimen exhibited a vascular-rich tumor, 24 × 24 × 19 mm in size, with deep ulceration in the duodenum. Histological examination showed uniform small round cells with central nuclei and a pale cytoplasm (glomus cell) with perivascular proliferation. Immunochemical studies showed that the tumor was positive for SMA and collagen type IV, and negative for C-Kit, CD34, desmin, and S100. We diagnosed the tumor as a GT of the duodenum.

**Conclusion:**

GTs of the duodenum are exceedingly rare, but should be considered in the differential diagnoses of duodenal submucosal lesions.

## Background

Glomus tumors (GTs) arise from the glomus bodies that act as skin thermoregulators, and are mostly found in the peripheral soft tissues at the dermal and subdermal subungual zones of fingers and toes [1]. Among the few reported cases, GTs arise from the gastrointestinal tract, and most of them originate from the stomach [2–5]. However, GTs arising from the duodenum are exceedingly rare. Here, we report a rare case of GT arising from the duodenum.

## Case presentation

### Patient

A 68-year-old man presented to his primary care physician with a complaint of abdominal pain. He had a medical history of reflux esophagitis and benign prostatic hyperplasia. Esophagogastroduodenoscopy was performed, which showed a submucosal mass in the second portion of the duodenum with central ulceration. Upon admission, his carcinoembryonic antigen (CEA) and carbohydrate antigen (CA) 19-9 levels were within normal limits.

### Dynamic computed tomography (CT)

**Fig. 1:**
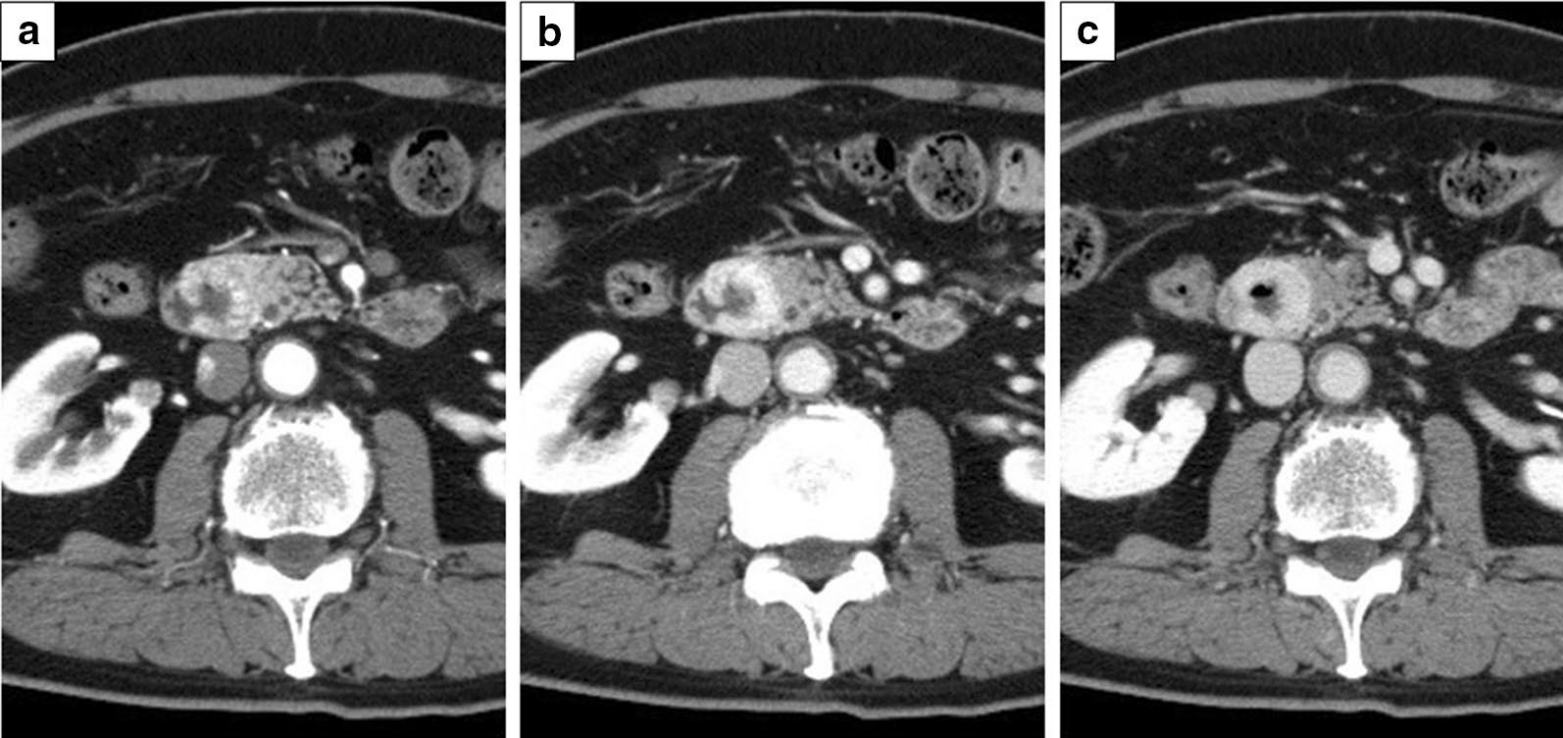
Dynamic computed tomography (CT) shows a circumscribed tumor measuring 26 mm in diameter with ulceration in the second portion of the duodenum. **a**, **b** On arterial (**a**) and portal phase (**b**) of contrast-enhanced CT, the mass reveals significant enhancement. **c** The mass shows persistent enhancement on the delay phase

Dynamic CT showed a circumscribed tumor measuring 26 mm in diameter in the second portion of the duodenum with ulceration. On the arterial and portal phase contrast-enhanced CT, the mass was greatly enhanced (Fig. 1a, b) with, persistent enhancement on the equilibrium phase (Fig. 1c). The mass was in contact with the pancreatic head, and pancreatic invasion was suspected. However, there was no dilatation of the central common bile duct and middle pancreatic duct.

### Magnetic resonance imaging (MRI)

**Fig. 2:**
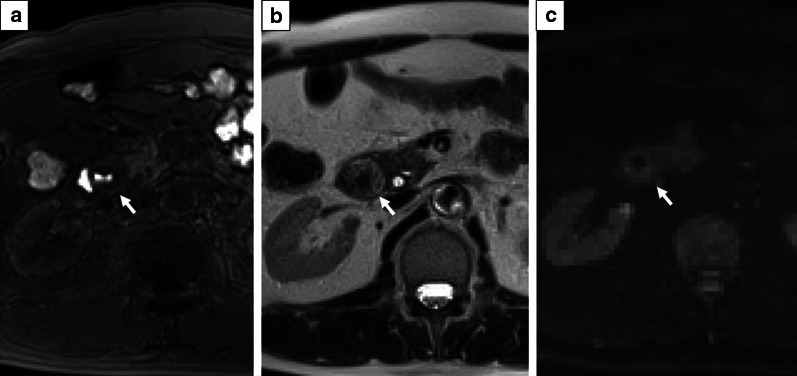
**a** The tumor revealed hypo-intensity on T1-weighted image. **b** The tumor shows a modestly high intensity on T2-weighted imaging. **c** The tumor shows a slightly high intensity on diffusion-weighted images

The T1-weighted image showed the tumor as a hypo-intensity (Fig. 2a), and the T2-weighted image showed a tumor with modestly high intensity (Fig. 2b). The tumor showed a slightly high intensity on diffusion-weighted imaging (DWI) (Fig. 2c), and the apparent diffusion coefficient (ADC) map displayed slight visual intensity.

### Esophagogastroduodenoscopy and endoscopic ultrasonography (EUS)

**Fig. 3:**
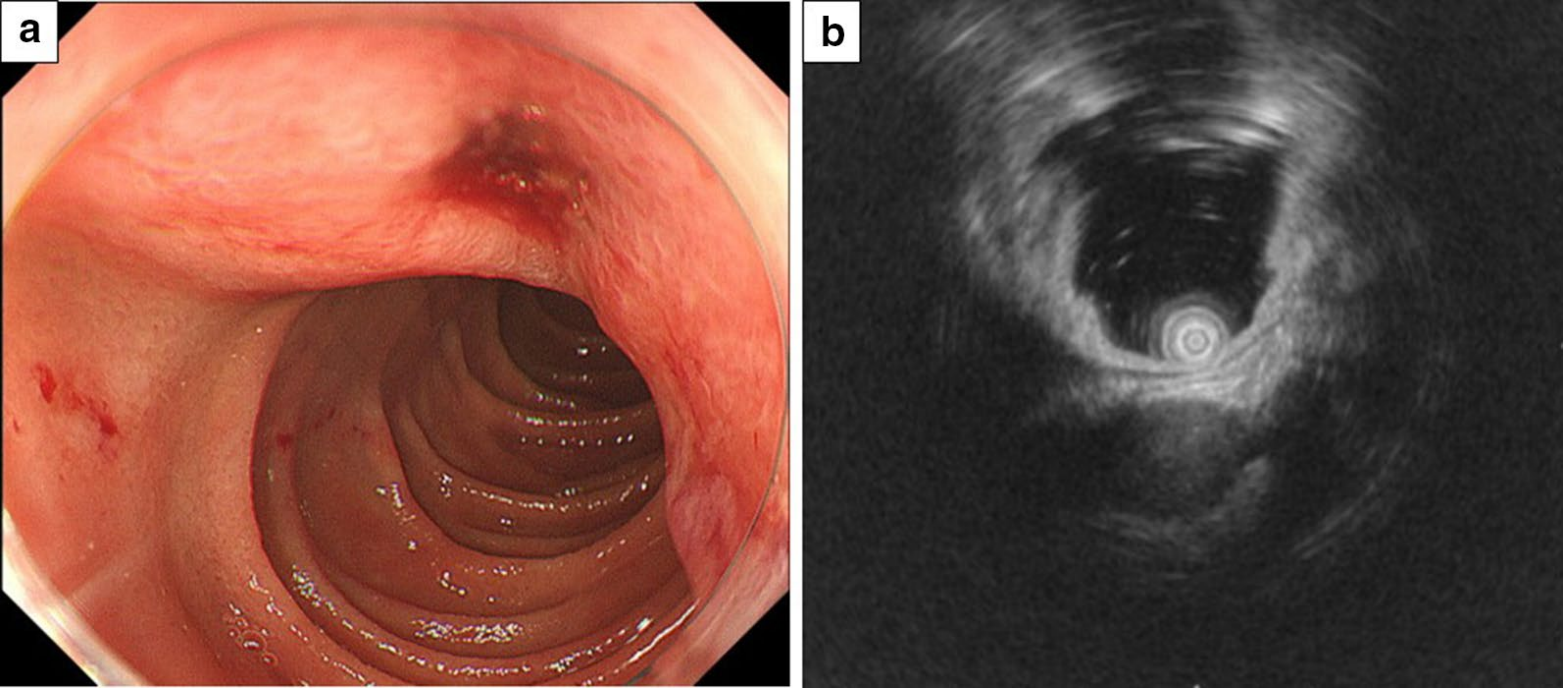
**a** Endoscopy shows a round, smooth, elevated mass in the second portion of the duodenum with central ulceration. **b** Endoscopic ultrasonography of the lesion confirms a hypoechoic mass arising from the fourth layer of the duodenal wall

Endoscopy showed a round, smooth, elevated mass in the second portion of the duodenum with central ulceration (Fig. 3a). Endoscopic ultrasonography of the lesion confirmed a hypoechoic mass arising from the fourth layer of the duodenal wall (Fig. 3b). A biopsy was performed from the central ulceration, but the cytological findings revealed no malignancy. Immunochemical studies were positive for smooth muscle actin (SMA) and negative for S100, C-Kit, and CD34.

Preoperatively, we diagnosed the tumor as a leiomyoma or gastrointestinal stromal tumor (GIST). Therefore, after informed consent was obtained, pancreatoduodenectomy (PD) was performed.

### Macroscopic findings

**Fig. 4:**
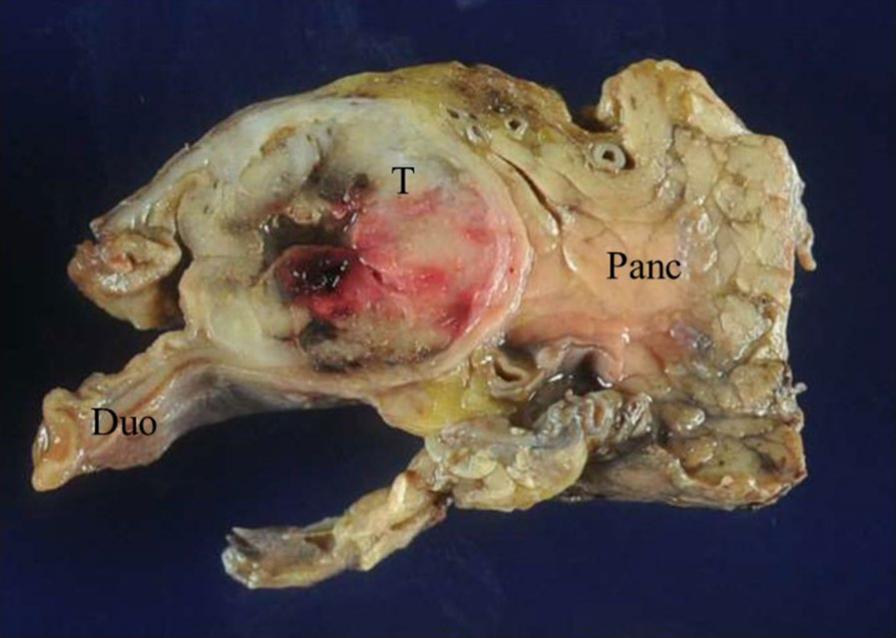
The resected specimen shows a yellowish-white tumor, 24 × 24 × 19 mm in diameter, arising from the submucosa of the duodenal wall with deep ulceration. *T* tumor, *Duo* duodenum, *Panc* pancreas

The resected specimen showed a yellowish-white tumor, 24 × 24 × 19 mm in diameter, arising from the submucosa of the duodenal wall with deep ulceration (Fig. 4). The tumor was located at the oral site from the papilla of Vater and showed transmural growth in the duodenal wall.

### Histological findings

**Fig. 5:**
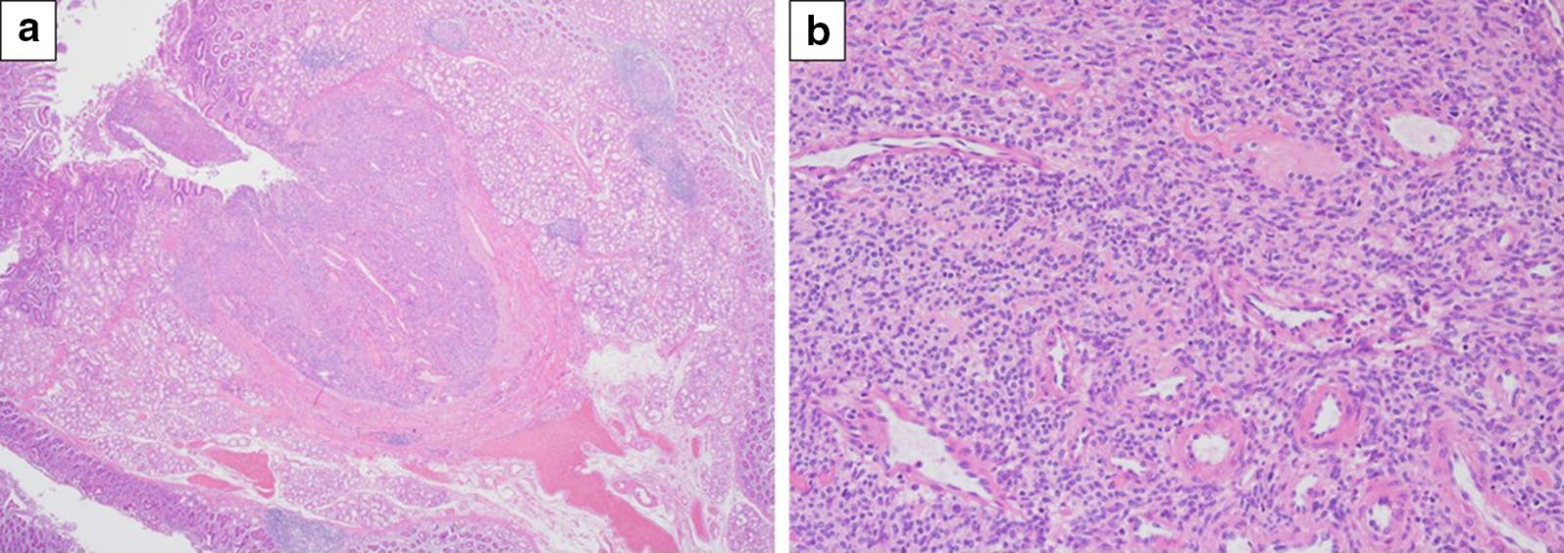
Histological examination shows uniform small round cells with central nuclei and a pale cytoplasm (glomus cell) with perivascular proliferation. **a** Hematoxylin and eosin stain (× 2). **b** Hematoxylin and eosin stain (× 20)

**Fig. 6:**
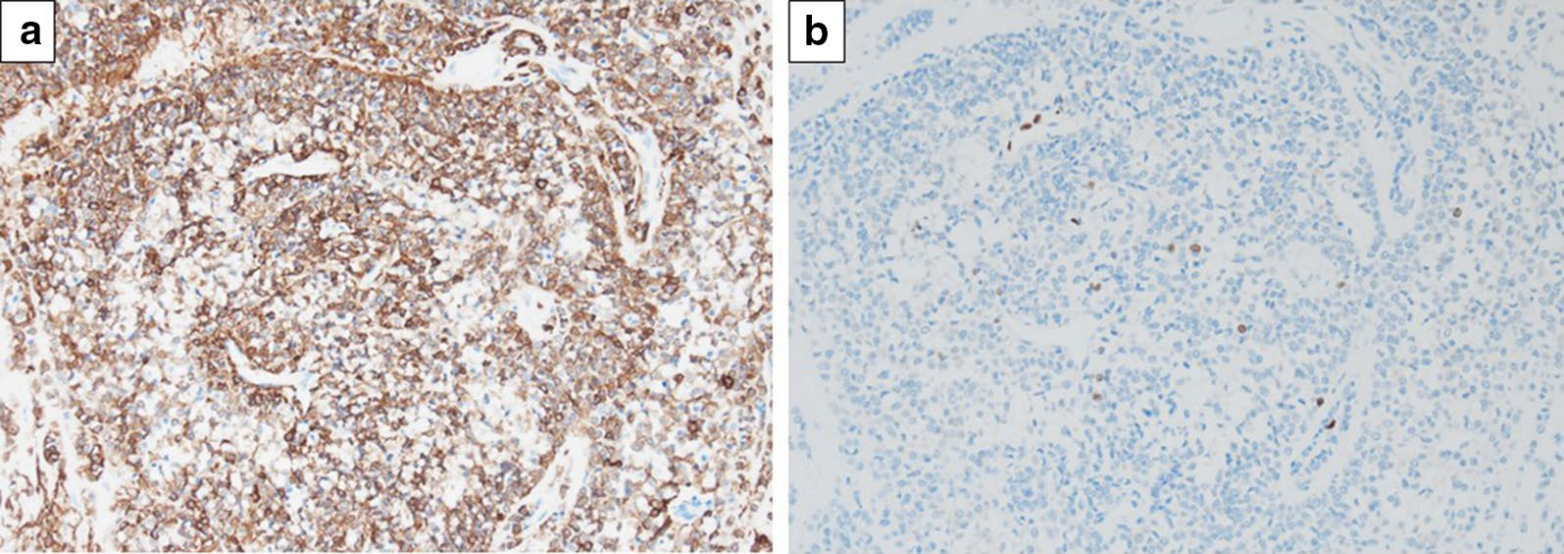
**a** Immunochemical studies shows that the tumor was positive for smooth muscle actin. **b** Ki-67 labeling index was 4% in the hot spot

The tumor was a vascular-rich tumor without capsular and deep ulceration (Fig. 5a). The lesion was shown by the nested or perivascular proliferation of mildly atypical cells with round-to-oval nuclei and eosinophilic cytoplasm (glomus cell), accompanied by prominent small blood vessels, hemorrhage and hyalinized stroma (Fig. 5b). The resected pancreas was free of tumor cells. No mitosis was observed. Immunochemical studies showed that the tumor was positive for SMA (Fig. 6a) and collagen type IV, and negative for C-Kit, CD34, desmin, and S100. The Ki-67 labeling index was 4% in the hot spot (Fig. 6b). As a result, the tumor was diagnosed as GT arising from the duodenum. There was no evidence of malignancy.

### Outcome

The patient was discharged from the hospital 38 days after the surgery. No sign of recurrence was found after a year of surgery.

## Discussion

GTs are rare benign neoplastic lesions arising from the glomus body, which is a component of skin thermoregulation [1]. GTs generally arise from the subungual zones of the fingers and toes; however, among the few reported cases, GTs have been reported to arise from the gastrointestinal tract and most of them from the stomach [3–5]. Gastric GT was first reported by Kay et al. in 1951[2] and is a rare disease accounting for approximately 1% of gastric mesenchymal tumors [5]. However, GT arising in the duodenum is exceedingly rare. In our search of the medical literature using PubMed with keywords “glomus tumor” and “duodenum”, we found only 7 cases reported in English literature, and 2 cases were reported in the Russian medical literature. A review of English literature, including our case, is presented in Table 1 [6–12]. There are 5 males and 2 females, with a median age of 57 (42–88) years. Most cases presented with gastrointestinal bleeding. In our literature review, there was no case that describes the medical history of GT of fingers and toes; we do not consider that patients with finger and toe GTs are at high risk for duodenal GTs. Since gastrointestinal GTs are rare, preoperative diagnosis is difficult. The differential diagnosis may include GIST, leiomyoma, neuroendocrine tumor (NET), lymphoma, ectopic pancreas, hemangioma, and secondary metastasis [13]. In our case, we preoperatively diagnosed GIST or leiomyoma. Because GTs have characteristic features of prominent vascular channels, they can be identified by dynamic CT. Hur BY et al. reported that a glomus tumor revealed strong enhancement on the arterial phase and prolonged enhancement on delayed phase (hemangioma-like enhancement) during dynamic CT [8]. GTs show more strong enhancement than other submucosal lesions in dynamic CT. Dynamic CT can contribute to the characterization of GTs. EUS is also useful for evaluating submucosal tumors. GTs show a round smooth mass usually arising from the third to fourth layer of the gastrointestinal wall, with varying internal echo patterns [14]. Furthermore, gastrointestinal GTs presented not only submucosal case, but have also been reported to bulge intraluminally to form a polypoid [3]. The usefulness of fluorodeoxyglucose (FDG)-positron emission tomography (PET)/CT in the GT is unclear. In some reports, FDG-PET/CT was useful for detecting lesion [15], but in the contrast, in some cases FDG-PET/CT did not show a significant uptake at the lesion [16]. Other benign gastric submucosal tumors were also reported to exhibit FDG avidity [17]; FDG-PET/CT may have a limited value in terms of the diagnosis of GTs.

**Table 1:** Literature review of case reports involving duodenum glomus tumor

Author Year	Age	Gender	Chief complaints	Initial diagnosis	Tumor size (cm)	Tumor location of the duodenum	Treatment	Prognosis
Jundi M 2004	46	Male	Melena	ND	2.3 × 1.5 × 1.5	Second portion,	Surgical resection (not described detail)	ND
Knackstedt C 2007	65	Male	Vomiting Upper gastrointestinal bleeding	Glomus tumor	ND	Duodenal bulb	Endoscopic mucosal resection	No signs of recurrence at 12 months
Shelton JH 2007	48	Female	Abdominal pain Melena	NET adenocarcinoma	3	Ampulla of Vater	PD	ND
Hur BY 2011	ND	ND	ND	ND	< 4.4	Duodenum (not described detail)	ND	ND
Tarangelo NP 2016	88	Female	Abdominal pain Melena Hematochezia	ND	2.1 × 1.4	Duodenal bulb	Artery embolization for bleeding	ND
Sadidoust A 2020	57	Male	Epigastric pain Dyspepsia	ND	1.2 × 0.7	Second portion	Endoscopic mucosal resection	No signs of recurrence at 48 months
Yoon J 2020	42	Male	Abdominal pain Melena	ND	2.5 × 1.5	Third portion	Laparoscopic wedge resection	No signs of recurrence at 18 months
Our case	68	Male	Abdominal pain	GIST Leiomyoma	2.4 × 2.4 × 1.9	Second portion	PD	No signs of recurrence at 12 months

*NET* neuroendocrine tumor, *GIST* gastrointestinal stromal tumor, *PD* pancreatoduodenectomy, *ND* not described

Histopathological findings showed uniform small basophilic round cells with central nuclei and pale eosinophilic cytoplasm (glomus cells) in alveolar hyperplasia around the small vessels. Immunohistochemical findings are useful for distinguishing GT from other subepithelial tumors. GT tumors are positive for SMA, vimentin, and collagen IV. In contrast, negative for C-Kit, CD34 (rarely positive), desmin, S100, and CD45 [3]. Most gastric GTs are benign, but malignant transformation has been reported in approximately 1% of all GTs [3]. Folpe et al. proposed the criteria for malignant GTs, including deep location and size greater than 2 cm, atypical mitotic figures, moderate-to-high nuclear grade, and ≥ 5 mitotic figures/50 HPF [18]. In our case, there were no mitotic figures, and the Ki-67 labeling index was 4% in the hot spot; these features were considered negative for malignancy. The prognosis of malignant GT of the duodenum remains enigmatic because of the scarcity of reported cases. There were no malignant GTs of the duodenum in our literature review. Although there is a report of malignant GT of the duodenum in the Russian literature, the case died from bleeding 23 days after the operation [19].

Treatment of duodenal GTs consists of endoscopic mucosal resection, laparoscopic wedge resection and PD (Table 1). Preoperatively, we suspected the tumor to be a leiomyoma based on immunochemical study of the tissue obtained from the tumor ulcer. However, since we did not perform EUS-fine needle aspiration (FNA), GIST was also suspected because of the possibility that the tissue was not adequate for diagnosis and imaging findings. Since the tumor was considered to have malignant potential (tumor size > 2 cm in diameter and with ulceration) and pancreatic invasion, we performed PD. Surgical treatment as a choice depends on the location and size of the tumor; in some reports, non-exposed endoscopic wall-inversion surgery (NEWS) and laparoscopic endoscopic cooperative surgery (LECS) have been used to resect gastrointestinal glomus tumor [20, 21].

## Conclusion

We present a rare case of GT in the duodenum. Duodenal GTs are difficult to distinguish from other submucosal lesions in the duodenum; however, immunohistochemical staining helps determine the correct diagnosis, and dynamic CT aids in preoperative diagnosis. Furthermore, GTs of the duodenum are exceedingly rare, but should be considered in the potential diagnosis of a duodenal submucosal lesion.

## Data Availability

Data will be made available from the corresponding author upon request.

## Abbreviations

GTs: Glomus tumors
CT: Computed tomography
EUS: Endoscopic ultrasound
SMA: Smooth muscle action
GIST: Gastrointestinal stromal tumor
PD: Pancreatoduodenectomy
CEA: Carcinoembryonic antigen
CA19-9: Carbohydrate antigen 19-9
MRI: Magnetic resonance imaging
EUS: Endoscopic ultrasonography
DWI: Diffusion-weighted images
ADC: Apparent diffusion coefficient
FDG: Fluorodeoxyglucose
PET: Positron emission tomography
FNA: Fine needle aspiration
NEWS: Non-exposed endoscopic wall-inversion surgery
LECS: Laparoscopic endoscopic cooperative surgery
